# On Diseases of the Skin

**Published:** 1857-11

**Authors:** 


ART. VIII.—*On Diseases of the Skin.* By ERASMUS WILSON, F.R.S. Fourth American, from the Fourth and Enlarged London Edition. Philadelphia: Blanchard & Lea. 8vo. pp. 649. 1857.

WE are happy to be able to introduce to our readers a new edition, the fourth, of Mr. Wilson's work on the "Diseases of the Skin," believing that it is the most complete and comprehensive treatise on the subject in the English language. It may now be regarded, indeed, as the standard work upon the subject. In preparing this edition, the author has made numerous additions, and has thus greatly enhanced its value. Among these additions the most important are, several new chapters on the classification, the general pathology, and the general therapeutics of cutaneous diseases, with remarks on furuncular eruptions, affections of the nails, burns, frostbite, parasitic animals, diseases of the sebiparous glands, scrofuloderma, and elephantiasis. He has also extended his account of the syphilitic eruptions, assigning to them a more important position, and arranging them according to their forms and the period of the poison. The volume closes with an appendix of selected formulæ, consisting, for the most part, of prescriptions which he has found most valuable in the treatment of skin diseases, to which frequent reference is made in the body of the work.

The work is accompanied by a series of plates, altogether nineteen in number, of which twelve are exquisitely colored. They represent nearly one hundred varieties of diseases, together with the normal anatomy of the skin, and are nearly all the size of nature.

It is not necessary to say anything in commendation of a work which has passed through so many editions as that of Mr. Wilson; nor anything respecting the necessity of studying a class of diseases so common as those of the skin. We believe it is generally acknowledged that the author is one of the ablest dermatologists in Europe, and this, surely, is enough honor for any man, however ambitious of fame. His practice, in this branch of medicine, is said to be very extensive, and it cannot be doubted that it must be remunerative in a city where there must be so much demand for this kind of knowledge and skill.

The American profession has not yet produced an original work on the diseases of the skin. We are aware that the late Dr. Worcester, of Cincinnati, published a treatise on that subject, but it was, as every one knows, chiefly a compilation from the works of Alibert, Rayer, Plumbe, Cazenave, and Schedel, and other writers. It was published when he was quite young, and has, we believe, long since been forgotten. The manual of Cazenave and Schedel has passed through several editions in this country, under the editorial supervision of Dr. Bulkley, of New York, one of the best authorities on skin diseases in the United States. Dr. Fisher, of Boston, published, many years ago, a beautiful and highly-creditable work on small-pox and varioloid; but, with these exceptions, nothing has been done in this country, so far as our information extends, for the department of dermatology. That we have, or might have, an abundance of opportunities for the study of the subject is unquestionable, and the only wonder is that it has not yet found any devotees. We venture to affirm that there is no department of the profession so little understood by the American practitioner as this; or one which, if properly pursued, holds out greater prospect of usefulness and fame.

